# A Novel Ethyl Formate Fumigation Strategy for Managing Yellow Tea Thrips (*Scirtothrips dorsalis*) in Greenhouse Cultivated Mangoes and Post-Harvest Fruits

**DOI:** 10.3390/insects14060568

**Published:** 2023-06-19

**Authors:** Kyeongnam Kim, Dongbin Kim, Soon Hwa Kwon, Gwang-Hyun Roh, Sangman Lee, Byung-Ho Lee, Sung-Eun Lee

**Affiliations:** 1 Institute of Quality and Safety Evaluation of Agricultural Product, Kyungpook National University, Daegu 41566, Republic of Korea; kn1188@knu.ac.kr (K.K.); dongbinkim@knu.ac.kr (D.K.); byungholee@hotmail.com (B.-H.L.); 2 Department of Applied Biosciences, Kyungpook National University, Daegu 41566, Republic of Korea; sangman@knu.ac.kr; 3 Citrus Research Institute, National Institute of Horticultural and Herbal Science, Seogwipo 63607, Republic of Korea; shkwonn@korea.kr; 4 Department of Plant Medicine and Institute of Agriculture & Life Sciences, Gyeongsang National University, Jinju 52828, Republic of Korea; ghroh@gnu.ac.kr

**Keywords:** pest management, climate change, tropical/subtropical mango, *Scirtothrips dorsalis*, scenario study

## Abstract

**Simple Summary:**

Climate change, along with increased demand for tropical fruits like mangoes, has led to an increase in greenhouse cultivation in South Korea, raising the risk of yellow tea thrips infestations. Our study explored ethyl formate fumigation as a safe, effective alternative to traditional pesticides in mango greenhouse cultivation and post-harvest storage. This novel fumigation method was successfully able to manage thrips without harming mango trees or fruits, offering an environmentally friendly pest management approach benefiting both farmers and consumers.

**Abstract:**

The effects of climate change and shifting consumer preferences for tropical/subtropical mango fruits have accelerated their greenhouse cultivation in South Korea, which has consequently exacerbated the risk of unexpected or exotic insect pest outbreaks. This study used the pest risk analysis (PRA) of greenhouse-cultivated mangoes provided by the Animal & Plant Quarantine Agency in Korea to evaluate the potential of ethyl formate (EF) fumigation as a new pest management strategy against the yellow tea thrips (*Scirtothrips dorsalis*), which is considered a surrogate pest in the thrips group according to the PRA. The efficacy and phytotoxicity of EF were evaluated in greenhouse-cultivated mango tree (Irwin variety) and post-harvest mango fruit scenarios. EF efficacy ranged from 6.25 to 6.89 g∙h/m³ for lethal concentration time (LCt)_50_ and from 17.10 to 18.18 g∙h/m³ for LCt_99_, indicating similar efficacy across both scenarios. Application of 10 g/m³ EF for 4 h at 23 °C could effectively control *S. dorsalis* (100% mortality) without causing phytotoxic damage to the greenhouse-cultivated mango trees, while post-harvest mango fruit fumigation with 15 g/m³ EF for 4 h at 10 °C showed potential for complete disinfestation of *S. dorsalis* without compromising fruit quality.

## 1. Introduction

Climate change predictions indicate that temperatures will have risen by 4 °C at the end of the 21st century, with the central region of the Korean Peninsula experiencing a warm temperate climate and the southern coast transitioning to a subtropical climate [1]. It is projected that 17% of the Korean Peninsula will become a subtropical climate zone [1]. Ten subtropical crops, including mango, passion fruit, dragon fruit, olive, papaya, atemoya, coffee, guava, feijoa, and banana, have been identified as suitable for the evolving Korean climatic environment [2]. The rapid development of horticultural facilities, such as greenhouses in Korea since the 1970s have enabled the cultivation of various crops [3].

Mango (*Mangifera indica*, Variety: Irwin) is a native tropical Asian crop and one of the most commercialized tropical fruits in the world. India is the largest global mango producer, followed by China, Thailand, Mexico, Pakistan, and Indonesia. Mango production in India accounts for about 38% of global mango production [4]. The cultivation area of tropical/subtropical crops in Korea has recently reached 292 ha, with an estimated 37 and 255 ha of fruit trees and vegetables, respectively [5]. Although the greenhouse production of the tropical/subtropical mangoes in Korea occupies a small area (25 ha), its cultivation is expected to increase due to their higher freshness index compared to the imported mango varieties, which have risen by 33% from 16,829 to 22,377 tons in 2020 and 2022, respectively [6]. Consequently, studies on the introduction and evaluation of subtropical/subtropical fruit tree performance, their impact assessment, and pest risk analysis (PRA) as potential hosts for exotic pests are crucial. PRA typically involves three stages, including initiation of the process, pest risk assessment, and pest risk management [7]. The emergence of insect pests associated with climate change is increasingly becoming a global environmental problem [8].

The yellow tea thrips (*Scirtothrips dorsalis* Hood) is a significant insect pest that affects citrus fruits in subtropical and tropical climates in Southeast Asia, and it is widely distributed throughout Asia, Australia, the Pacific coast islands, and parts of South Africa [9]. Minor crop damage caused by *S. dorsalis* has been reported in the Korean Jeju Island; however, its infection and severe damage to citrus orchards has been steadily increasing and spreading to other areas since 2007 [10]. The introduction of *S. dorsalis* populations into orchards is thought to originate from the surrounding wild host plants, leading to crop damage even after multiple pesticide treatments [11]. Controlling the thrips population with general insecticides is difficult due to their varied habitat, type, and tendency to effectively hide under plant leaves [12]. Moreover, their control is hindered by the development of insecticide-resistant strains in domestic cultivation areas. Therefore, selecting effective fumigants for their control is necessary [13].

According to the PRA for tropical/subtropical fruit cultivation, thrips such as *S. dorsalis* are classified as high-risk insect pests in cultivation areas due to their host preference, the potential for introduction, and establishment. Therefore, effective and safe disinfestation methods are needed to overcome pesticide resistance and potential residual toxicities associated with exotic thrips management in the future. Fumigation is commonly used to disinfect stored grain and soil pathogens, and for nematode control, as well as for quarantine disinfestation [14,15,16]. It is an effective and economical method for broad-spectrum control of insect pests in confined or semi-confined spaces. Ethyl formate (EF) has actively been studied as an alternative fumigant in Korea that is safer than methyl bromide (MB), which has ozone depletion properties and high toxicity to farm workers [17]. EF is routinely used to disinfect various commodities, such as numerous types of fruits, vegetables, nursery plants, and other non-food commodities [18,19,20,21,22,23,24,25,26,27,28,29,30,31,32]. The use of EF as a new management strategy instead of conventional pesticides to control agricultural pests in greenhouses, such as the pesticide-resistant whiteflies, has previously been reported [32,33].

In this study, the efficacy of EF as a disinfestation treatment was evaluated against *S. dorsalis*, a surrogate for the exotic thrips in tropical mango cultivation, with the following objectives: (1) to assess the efficacy of EF against *S. dorsalis*, (2) to investigate the performance of greenhouse EF fumigation in controlling *S. dorsalis* under cultivation conditions (>23 °C), to evaluate EF phytotoxic effects on mango trees and human safety, and (3) to examine the effectiveness of EF in post-harvest pest management in fruit stored at >10 °C, as well as its sorption and phytotoxic effects on mango fruits.

## 2. Materials and Methods

### 2.1. Fumigants

Liquid EF (99%, Fumate^TM^) was supplied by Safefume Inc. (Daegu, Republic of Korea) For lab fumigation (0.8 m^3^, 0.8 × 0.7 × 1.4 m) of post-harvest mango fruits, liquid EF was vaporized using a prototype EF vaporizer (Safefume Inc., Daegu, Republic of Korea), and mixed with nitrogen carrier gas to form a non-flammable EF fumigant formulation. For greenhouse fumigation of mango trees (340 m^3^, 25 × 4 × 3.4 m), liquid EF was vaporized using a newly developed airless pump for EF spraying with a micro-fine nozzle that enables it to be vaporized under natural temperature conditions in a greenhouse (Provided Safefume Inc., Daegu, Republic of Korea).

### 2.2. Insects

*Scirtothrips dorsalis* were collected in citrus orchards located in Jeju Island, Republic of Korea, and fed on citrus leaves at 25 ± 1 °C, 60% relative humidity (RH), and 16:8 h light/dark photoperiod.

### 2.3. Efficacy of EF against S. dorsalis during Greenhouse Mango Tree Cultivation and Post-Harvest Mango Fruit Storage

The EF fumigation against *S. dorsalis* was performed using 6.9 L desiccators and the magnetic fan was used at the bottom of each desiccator for inner air circulation. Samples in insect breeding dishes (4.5 cm diameter) were placed inside the desiccators with inoculated *S. dorsalis*. After sealing each desiccator, EF was injected in liquid form in the range of 0.5, 1, 2, 4, 8, and 10.0 g/m^3^ using a gas-tight syringe (SGE Analytical Science, Melbourne, VIC, Australia), which was calculated based on the equation reported by [34]. The desiccators treated with EF were kept for 4 h at 23 °C and 11 °C for the cultivation and post-harvest storage scenarios, respectively. The fumigated *S. dorsalis* insects were then transferred to the insect rearing room and kept under 25 ± 2 °C and 75% ± 5% RH conditions, and adult mortality was analyzed three days after fumigation. At least 20 adult *S. dorsalis* were used per replication, and all experiments were performed in three replicates with a control group.

### 2.4. Post-Harvest Mango Fruit Storage Scenario

#### 2.4.1. Evaluation of EF Sorption in Mango Fruits

Analysis of EF sorption in mango fruits was conducted in a 0.8 m^3^ fumigation chamber with a 30% (*w*/*v*) loading ratio of mango fruits. The fruits were fumigated with 15 and 30 g/m^3^ EF for 4 h at 11 °C. For the determination of EF gas concentrations of each desiccator, gas was collected using 1 L Tedlar bag (SKC Inc., Dorset, UK) and vacuum pump (Gast, IDEX corp., Benton harbor, MI, USA) at the four time points (0.1, 1.0, 2.0, and 4.0 h) after EF fumigation. In addition, gas chromatography equipped with a flame ionization detector (GC-FID, a Shimadzu-GC 17A, Shimadzu, Kyoto, Japan) was used with a DB5-MS Column (30 m × 0.25 mm i.d., 0.25 µm, J & W Scientific, Folsom, CA, USA). The oven temperature was set at 100 °C, while the injector and detector temperatures were set at 250 and 280 °C, respectively. Helium carrier gas was supplied at a flow rate of 1.5 mL/min. The standard curve of EF was calculated with the peak area of a series in the range of 0.1–15 g/m^3^. Sorption rate of EF was calculated as ratio (C/C_0_), where the C (EF concentration was determined at one of the time intervals) and the C_0_ (EF concentration at 0.1 h).

#### 2.4.2. Phytotoxic Assessments of Mango Fruits

Fruits were supplied with 15 and 30 g/m^3^ EF, which was held for 4 h at 11 °C. Phytotoxicity was analyzed 7 days after post-harvest storage at (11 ± 1 °C) by assessing firmness, sugar content, and color change, and weight loss after 4 h-EF-fumigation with 15 and 30 g/m^3^. Five fruit samples from each group were used to measure the firmness in triplicates using a fruit firmness tester (Digital fruit firmness tester with an 8 mm steel plunger, TR Turoni, Forlì, Italy). To measure the soluble sugar contents after EF fumigation, each fruit filtrate (0.5 mL) was dropped on a portable refractometer (Hand refractometer ATC-1E, Atago Co., Ltd., Tokyo, Japan); then, the sugar contents of five fruits per measurement recorded from the refractometer scale reader screen. A colorimeter (TES 135A, Electrical & Electronic Corp., Taipei, Taiwan), which expresses color in Hunter L*, a*, and b* values, was used to measure fruit color change. Fruit weight loss was expressed as a ratio between the weight before treatment and the weight after seven days of fumigation.

### 2.5. Fumigation of the Greenhouse-Cultivated Mango Tree Scenario

#### 2.5.1. Evaluation of EF Sorption in Greenhouse Mango Trees

Fumigation of mango trees with 10 g/m^3^ EF for 4 h at 23 °C was conducted in 340 m^3^ greenhouse located in Sancheong-gun, Republic of Korea. Trees were inoculated with *S. dorsalis* grown on an insect breeding dish (4.5 cm diameter), which was located at several sites in the greenhouse. Gas sampling lines were set at the three top, middle, and bottom points of the greenhouse to monitor EF concentration. Collection of gas samples was performed at 0, 1, 2, and 4 h time intervals for measurement of EF concentration using gas chromatography-flame ionization detector (GC-FID). A vaporizer was used to supply 10 g/m^3^ EF in the greenhouse for 4 h at 23 ± 2 °C. After 4 h fumigation, the container was opened and ventilated for 1 h to decrease the gas concentration, followed by measurement of the desorption rate using GC-FID. Phytotoxicity was assessed on mango trees, while adult *S. dorsalis* mortality was evaluated using a microscope after seven days of fumigation.

#### 2.5.2. Phytotoxicity Assessment of Mango Trees

The damage indexes were evaluated with the following scale: 0, 1, 2, 3, and 4, representing no leaf damage, <5% of total leaves/plant dropped/browned/shriveled, 5–25% leaves affected, 25–50% leaves affected, and >50% leaves affected, respectively. Chlorophyll content was measured the 10 points of mango tree leaves using SPAD-502 Plus (Minolta, Tokyo, Japan). The color changes were calculated as Hue value ([L^2^ + a^2^ + b^2^]/2) from the values from ten different mango trees after fumigation.

#### 2.5.3. Assessment of Worker Safety in the Greenhouse

Gas concentration in the greenhouse was measured using a portable gas analyzer (RAE Systems Minirae 3000, JJS Technical Services, Schaumburg, IL, USA) to assess worker safety during ventilation at different time intervals after the end of fumigation experiments.

### 2.6. Statistical Analysis

The toxicological response of *S. dorsalis* against EF dosage was analyzed using a Probit analysis. Assessment of all data-related quality parameters, such as weight loss, firmness, sugar content, and surface color change were calculated using Proc Univariate in SAS (SAS Institute, Cary, NC, USA). To analyze the phytotoxic damage of EF fumigation on mangoes, a one-way ANOVA was performed in SAS (ver. 9.4; SAS Institute Inc., 1998) based on Tukey’s Studentized Range (HSD) test at *p* = 0.05.

## 3. Results

### 3.1. Efficacy of 4 h EF Fumigation on S. dorsalis in Two Different Scenarios

**Figure 1:**
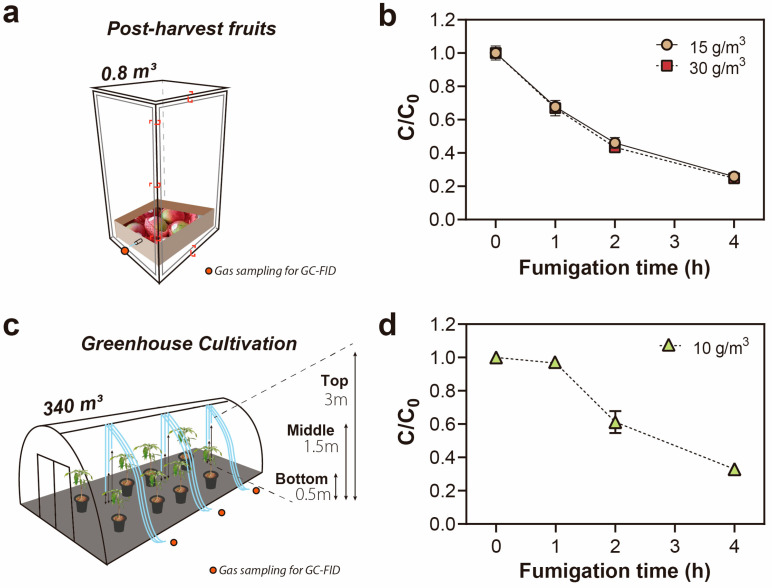
Comparison of the ethyl formate (EF) sorption rate in two different scenarios. (**a**,**b**) Post-harvest storage scenario (15 or 30 g/m^3^ EF for 4 h at 11 ± 1 °C, filling ratio: 30%); (**c**,**d**) Greenhouse cultivation scenario (10 g/m^3^ EF for 4 h at 23 ± 1 °C).

The LCt_50_ and LCt_99_ values of EF were 6.89 and 18.18 g h/m^3^ for adult *S. dorsalis*, with a fitted slope value of 5.52 ± 0.4 after 4 h fumigation at 11 °C in the post-harvest storage scenario (Table 1). The EF concentration in the fumigation chamber decreased during the 4 h fumigation period with 30% (*w*/*v*) loading ratios of mango fruits (Figure 1a). Mango fruits with a 30% loading ratio (*w*/*v*) and an EF applied dose target at the LCt_99_ level exhibited sorption of about 80% at the end of the fumigation treatment (Figure 1b).

For the cultivation scenario at 23 °C, LCt_50_ and LCt_99_ values of EF were 6.25 and 17.10 g h/m^3^ for the adult *S. dorsalis*, with a fitted slope value of 5.32 ± 0.4 after 4 h fumigation (Table 1). During the 4 h EF fumigation of cultivated trees at 23 °C, EF concentration systematically decreased throughout the fumigation period due to its sorption to mango trees and soil to a final 35% concentration of its initial dose (Figure 1c,d).

### 3.2. Phytotoxic Assessment of Post-Harvest Mango Fruit Storage

**Figure 2:**
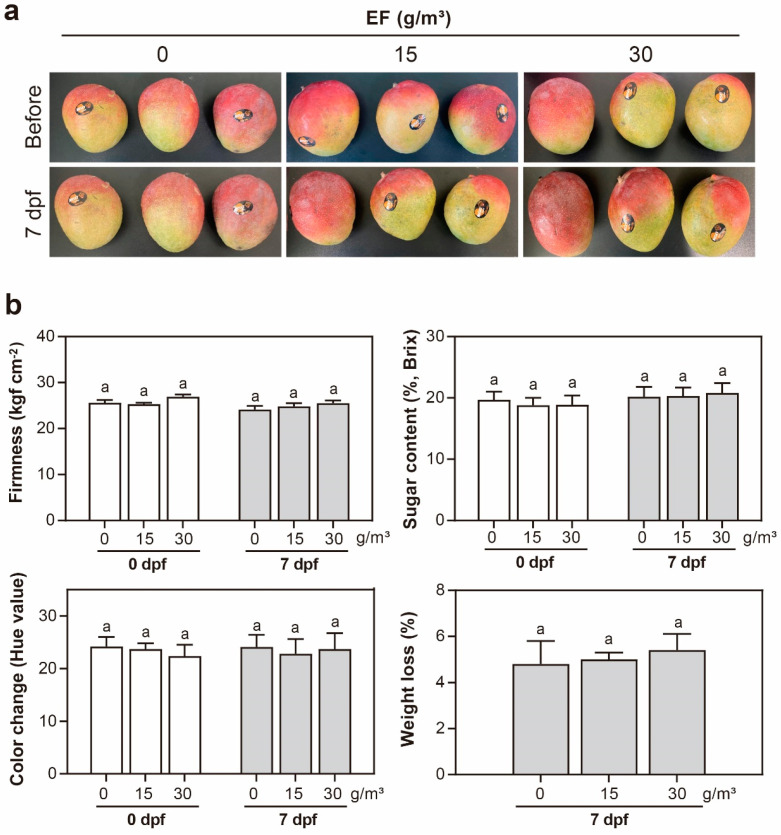
Phytotoxic assessment of post-harvest mango fruits after EF fumigation under the storage scenario. EF fumigation at 15 and 30 g/m^3^ concentration for 4 h at 11 ± 1 °C was applied, then effects were observed at 0 and 7 days post fumigation (dpf). (Ct values; 25.8 g h/m^3^ for EF 15 g/m^3^, 41.7 g h/m^3^ for EF 30 g/m^3^). (**a**) Photographs of mango fruits before and after EF fumigation; (**b**) comparison of phytotoxic indices. The same letters are not significantly different at the 5% level.

No phytotoxic effects were observed in the post-harvest mango fruits (Variety; Irwin) treated with 15 and 30 g/m^3^ EF until 7 days after fumigation, with the two concentrations showing Ct values of 25.8 and 41.7 g∙h/m^3^, respectively (Figure 2a). Means of firmness in the control and 15 or 30 g/m^3^ EF treatment at 7 days post fumigation were 24.1, 24.8, and 25.5 kgf cm^−2^, respectively (Figure 2b). No significant difference was observed in fruit firmness (kgf cm^−2^), 0.38% LSD (*p* = 0.05). The mean fruit sugar contents in the control and 15 or 30 g/m^3^ EF treatments were 20.2, 20.3, and 20.8%, respectively (Figure 2b).

No significant difference (0.38% LSD, *p* = 0.05) in sugar content (%, brix) was observed. Similarly, the means of fruit color changes in the control and 15 or 30 g/m^3^ EF treatments were 24.1, 22.8, and 23.7, respectively, indicating no significant difference in the color change (hue values) based on LSD (0.38%, *p* = 0.05) (Figure 2b). Moreover, the mean fruit weight in the control and 15 or 30 g/m^3^ EF treatments were 4.8, 5.0, and 5.4%, respectively, which suggested no significant difference in weight loss (%) based on LSD (0.38%, *p* = 0.05). Overall, these results showed no evidence of phytotoxic damage on firmness, sugar content, color change, and weight loss during post-harvest storage of mango fruits after 4 h EF fumigation (Figure 2b).

### 3.3. EF Concentration during/after Fumigation and Its Phytotoxic Effects in Cultivation Mango Trees

The concentration of EF at time intervals during each fumigation cycle is shown in Table 2. The Ct values of EF in mango trees at the top, middle, and bottom greenhouse locations were 22.7 ± 0.3, 20.6 ± 0.3, and 20.4 ± 0.1 g∙h/m^3^, respectively. The EF concentration at the top location of the greenhouse was constantly higher than at the middle and bottom locations throughout the 4 h treatment period (Table 2). There was an approximately 1.1–1.2-fold difference in the Ct values between the top and bottom locations, which did not affect the target Ct value for controlling *S. dorsalis*. Fumigation with 10 g/m^3^ EF successfully caused 100% mortality of *S. dorsalis* adults in the greenhouse-cultivated mango plants (Table 2).

**Figure 3:**
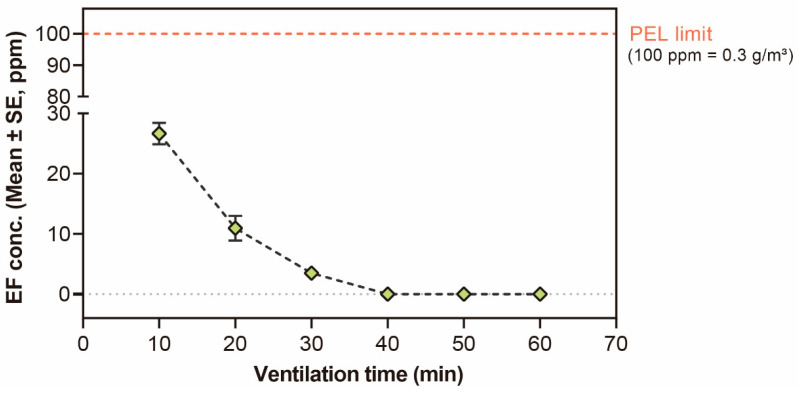
Evaluation of ethyl formate (EF) concentration safety in the greenhouse. EF levels in the greenhouse after time interval natural ventilation at 4 h post fumigation (23 ± 1 °C, RH: 70–90%; the permissible exposure limit of EF is 100 ppm).

In the assessment of worker safety, EF concentration during the greenhouse ventilation process showed a rapid decrease to less than 100 ppm (EF at 100 ppm is the acceptable exposure level in Korea) within 10 min, but more than 1 h was still needed to lower its potential hazardous conditions, which could cause unexpected acute inhalation risk in the greenhouse (Figure 3).

**Figure 4:**
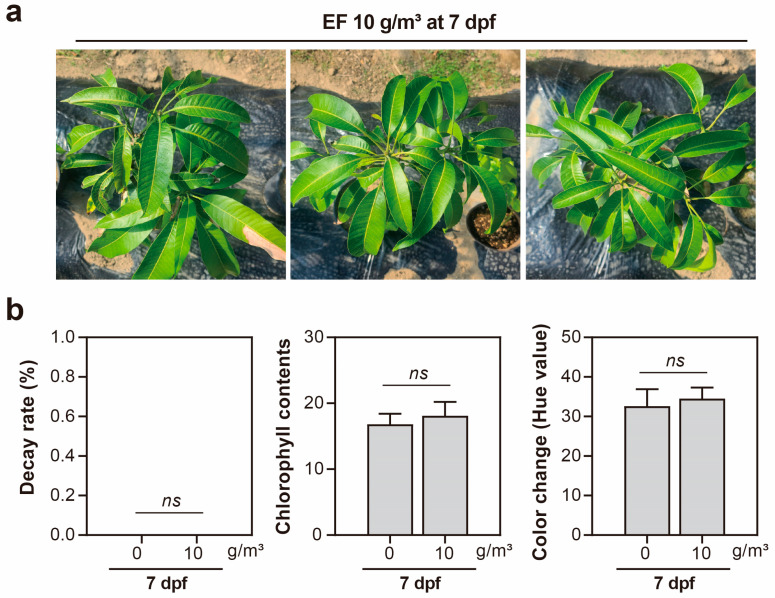
Phytotoxic assessments of mango trees after fumigation with 10 g/m^3^ EF for 4 h under the cultivation scenario at 23 ± 1 °C (Ct values: 21.23 ± 0.7 g h/m^3^). (**a**) Photographs of mango trees at 7-days post fumigation (dpf). (**b**) Comparison of phytotoxic indices on mango tree leaves. *ns*, Not significant at the 5% level.

Chlorophyll content was quantified in the greenhouse mango trees after one-week treatment to assess EF phytotoxicity (Figure 4). Mean chlorophyll contents in the untreated and 10 g/m^3^ EF treatment trees were 16.8 and 18.1, respectively, which indicated no statistical difference in chlorophyll contents (0.38% LSD, *p* = 0.05) between treatments. Color changes in the cultivation scenario were investigated after 1 week. No significant difference (0.38% LSD, *p* = 0.05) in the means of color change was observed between the untreated (32.6) and 10 g/m^3^ EF treated (34.5) greenhouse mango trees after one-week fumigation. Overall, these results demonstrate that continuous EF fumigation could control *S. dorsalis* (100% mortality) without residual or phytotoxic effects in greenhouse-cultivated mangoes (Figure 3 and Figure 4).

## 4. Discussion

### 4.1. Efficacy of Various Thrips Using Fumigant and Other Pesticides

Various traditional insecticides such as abamectin, acetamiprid, and spinosad have been used to manage thrips (*Thysanoptera*: *Thrididae*) [35]. However, controlling thrips can be highly challenging due to their life cycle characteristics, including a pupa stage in soil, their slender bodies, and their rapid development of resistance [36]. In particular, *S. dorsalis*, the target pest of this study, is known to infest a wide range of over 225 host plants, including mango, avocado, strawberry, and chili pepper [37]. The slender bodies and elusive nature of thrips not only reduce the effectiveness of traditional pesticides by decreasing their contact amount, but also necessitate increased pesticide usage [35,36]. Given these challenges, gas-type fumigation methods like EF can be highly effective, as they permeate the environment, reaching thrips regardless of their attempts to evade or escape.

Existing studies have highlighted the efficacy of EF against *S. dorsalis* and various other thrips species. For instance, the Probit 9 values for adult bean thrips (*Caliothrips fasciatus* Pergande) treated with EF fumigation for one hour at 5, 10, and 15 °C were 12.99, 17.05, and 14.32 g h/m^3^, respectively, showcasing successful pest control without damaging the host plant, citrus [38]. Aharoni et al. (1980) reported the successful application of EF under vacuum conditions against western flower thrips (*Frankliniella occidentalis*) on post-harvest strawberries, achieving full mortality without any negative impacts on the strawberries’ taste, odor, or decay rate [39]. Similarly, other studies have demonstrated that one-hour EF fumigation at 1 and 20 °C yielded Probit 9 values of 7.4 and 38.1 g h/m^3^, respectively [40]. Simpson et al. (2007) found that *F. occidentalis* could be controlled at 10% CO_2_ with 6.51 g/m^3^ of EF [18]. In another study, it was reported that an EF concentration of 8 g/m^3^ for over 30 min at 20 °C was effective against adult onion thrips (*Thrips tabaci* Lindeman) [41].

### 4.2. Phytotoxicity of EF towards Cultivated Mango Trees and Post-Harvest Fruits

The potential for ethyl formate (EF) to cause phytotoxicity has previously been reported in the leaves of imported foliage nursery plants [26]. However, numerous studies have shown that EF exhibits no phytotoxic effects on a variety of fruits, such as yellow melon, citrus, pineapples, and grapes [17,18,22,32]. It has generally been found that EF fumigation impacts leaves more significantly than fruits [17,22,26]. Interestingly, in this study, mango leaves displayed no phytotoxic effects even when exposed to relatively high Ct values of EF (Figure 4). The robust resistance to EF-induced phytotoxicity observed in mango trees might be attributed to their leaf phenotypes. These leaves often possess relatively thick cuticles, which facilitate water conservation and offer resistance to the typical hot and dry conditions found in tropical climates [42]. This observation suggested that EF fumigation could be a viable, non-phytotoxic method for the treatment of mango fruit post harvest and during storage.

Additionally, in this study, we assessed phytotoxic effects, including changes in firmness, sugar content, and color of the mango fruits (Figure 2). It is important to note, however, that mangoes are rich in numerous bioactive compounds like phenolic compounds, beta-carotene, and vitamin C. In particular, mangiferin, a dominant active phenolic component in mango, has demonstrated potential anti-cancer, anti-bacterial, anti-atherosclerotic, anti-allergic, anti-inflammatory, anti-obesity, analgesic, and immune-regulatory properties [43,44]. Therefore, a valuable direction for future research would be to analyze the alterations in these bioactive compounds in mangoes pre and post EF fumigation.

### 4.3. Greenhouse Application of EF Fumigation

EF is a naturally occurring volatile compound that rapidly evaporates upon exposure to air due to its relatively low boiling point of approximately 54 °C. This characteristic makes it well-suited for application in greenhouses. In the scenario of cultivation in mango tree in the greenhouse, the LCt_99_ value for controlling *S. dorsalis* was 17.10 (14.83–20.63) g h/m^3^ (Table 1), the target Ct value over 20 g h/m^3^ was successfully reached in the greenhouse (Table 2). The concentration of EF in three gas sampling points reached the target Ct value. Still, observations on the Ct value reveal that the final EF concentration was significantly higher at the top of the greenhouse than the middle and bottom, which aligns with previous research findings on controlling *Bemisia tabaci* [32]. The reduction of EF concentration at the bottom of the greenhouse is likely due to absorption by the plants and soil present. On the other hand, a safe concentration in the atmosphere of greenhouse air was observed 10 min after opening below Permissible Exposure Limit (PEL) limit (Figure 3), and the concentration of EF in the air was not completely measured after 1 h in this study and a previous study [32]. This novel EF fumigation strategy was suggested for managing *S. dordalis* in greenhouse-cultivated mangos and post-harvest mango fruits without phytotoxicity on both fruits and leaves and residual issues.

## 5. Conclusions

In summary, fumigation with 10 g/m^3^ EF for 4 h at 23 °C under the cultivation scenario caused 100% *S. dorsalis* mortality on greenhouse mango trees with no phytotoxic damage at levels higher than the EF LCt_99_ value (17.10 g h/m^3^ for 4 h at 23 °C). Mango fruits treated with 15 and 30 g/m^3^ EF for 4 h at 11 ± 1 °C under the post-harvest storage scenarios showed no phytotoxic damage at higher levels than the EF LCt_99_ value (18.18 g h/m^3^ for 4 h at 11 °C). We speculate that EF fumigation technology, which is currently replacing usage of MB as QPS in Korea, is potentially an effective fumigant against *S. dorsalis* that can be used to control unexpected outbreaks of exotic pests in greenhouse-cultivated plants or during post-harvest fruit storage without altering their quality.

## Figures and Tables

**Table 1:** The efficacy of EF fumigation for 4 h against *Scirtothrips dorsalis* adults in different scenarios.

Scenarios	Temp. (°C)	^1^ LCt_50_ (95% CL, g h/m^3^)	LCt_99_ (95% CL, g h/m^3^)	Slope ± SE	*df*	*X^2^*
Post-harvest mango fruits	11	6.89 (6.48–7.33)	18.18 (15.91–21.62)	5.52 ± 0.4	25	27.55
Cultivation in mango tree in greenhouse	23	6.25 (5.86–6.67)	17.10 (14.83–20.63)	5.32 ± 0.4	8	29.42

^1^ Lethal concentration time.

**Table 2:** Concentration, concentration–time (Ct) values at different greenhouse locations, and ethyl formate (EF) efficacy against *Scirtothrips dorsalis* adults.

Applied Dose (g/m^3^)	Exposure Time (h)	EF Concentration (Mean ± SE, g/m^3^)			Mortality (Mean ± SE, %)
		Top	Middle	Bottom	
10	0.1	8.3 ± 0.1	7.9 ± 0.1	7.8 ± 0.1	100 ± 0.0
	1.0	7.9 ± 0.1	7.8 ± 0.1	7.5 ± 0.1	
	2.0	5.5 ± 0.2	4.5 ± 0.1	4.7 ± 0.1	
	4.0	2.9 ± 0.1	2.5 ± 0.0	2.5 ± 0.1	
Ct values (Mean ± SE, g h/m^3^)		22.7 ± 0.3 ^a,†^	20.6 ± 0.3 ^b^	20.4 ± 0.1 ^b^	

^†^ The same letters are not significantly different at the 5% level (a > b).

## Data Availability

All the data generated in this work were provided in the article.

## References

[1] Ji S.T., Youm J.W., Yoo J.Y. (2018). A feasibility study on the cultivation of tropical fruit in Korea: Focused on mango. J. Korea Acad. Ind. Coop. Soc..

[2] Kang K.M., Choi Y.E., Kim Y.J., Min S.J., Choi D.S., Kim K.Y., Lee D.Y. (2021). The classification of climate types and the delineation of their climatic characteristics using new nomals (1991–2020) in the Republic Korea. J. Clim. Res..

[3] Choi M.K., Yun S.W., Kim H.T., Lee S.Y., Yoon Y.C. (2014). Field survey on the maintenance status of greenhouse in Korea. Prot. Hortic. Plant Fact..

[4] Yadav D., Singh S.P. (2017). Mango: History origin and distribution. J. Pharmacogn. Phytochem..

[5] Kim C.Y., Kim Y.H., Han S.H., Ko H.C. (2019). Current situation and prospects on the cultivation program of tropical and subtropical crops in Korea. Korean J. Plant Res..

[6] Animal and Plant Quarantine Agency (AQPA). https://okminwon.pqis.go.kr/minwon/information/statistics.html?statsType=103&frYear=2021&frMonth=01&toYear=2021&toMonth=12&trnType=IN&metType=hwa&itemCd=22150532&itemNm=%EB%A7%9D%EA%B3%A0&x=43&y=16.

[7] IPPC (2012). International Standards for Phytosanitary Measures, Publication No. 5: Glossary of Phytosanitary Terms.

[8] Hulme P.E. (2009). Trade, transport and trouble: Managing invasive species pathway in an era of globalization. J. Appl. Ecol..

[9] Smith D., Pena J.E., Pena J.E., Sharp J.L., Wysoki M. (2002). Tropical citrus pests. Tropical Fruit Pests and Pollinators, Biology, Economic Importance, Natural Enemies and Control.

[10] Hyun J.W., Hwang R.Y., Lee K.S., Song J.H., Lee P.H., Kwon H.M., Hyun D.H., Kim K.S. (2012). Seasonal occurrence of yellow tea thrips, *Scirtothrips dorsalis* Hood (*Thysanoptera*: *Thripidae*) in citrus orchards and its damage symptoms on citrus fruits. Korean J. Appl. Entomol..

[11] Masui S. (2008). Estimation of the immigration time of *Scirtothrips dorsalis* Hood (*Thysanoptera: Thripidae*) adults in citrus orchards as a function of the total effective temperature. Appl. Entomol. Zool..

[12] Yu J.S., Kim J.I., Kim G.H. (2002). Insecticide susceptibilities of rose field-collected populations of western flower thrips, *Frankliniella occidentalis* in Korea. Korean J. Pest. Sci..

[13] Kyung Y.J., Kim H.K., Lee J.S., Kim B.S., Yang J.O., Lee B.H., Koo H.N., Kim G.H. (2018). Efficacy and phytotoxicity of phosphine as fumigants for *Frankliniella occidentalis* (*Thysanoptera*: *Thripidae*) on asparagus. J. Econ. Entomol..

[14] Bell C.H. (2000). Fumigation in the 21st century. Crop Prot..

[15] Rambeau M.B., Benitez D.P., Dupuis S., Ducom P., Donahaye E.J., Navarro S., Leesch J.G. (2001). Hydrogen cyanide as an immediate alternative to methyl bromide for structural fumigations. Proceedings of the International Conference Controlled Atmosphere and Fumigation in Stored Products.

[16] Campbell J.F., Toews M.D., Arthur F.H., Arbogast R.T. (2010). Long-term monitoring of *Tribolium castaneum* in two flour mills: Seasonal patterns and impact of fumigation. J. Econ. Entomol..

[17] Park M.G., Lee B.H., Yang J.O., Kim B.S., Roh G.H., Kendra P.E., Cha D.H. (2021). Ethyl formate as a methyl bromide alternative for fumigation of citrus: Efficacy, fruit quality, and workplace safety. J. Econ. Entomol..

[18] Simpson T., Bikoba V., Tipping C., Mitcham E.J. (2007). Ethyl formate as a postharvest fumigant for selected pests of table grapes. J. Econ. Entomol..

[19] Misumi T., Ogawa N., Yamada K., Shukuya T. (2013). Susceptibilities of five species of scales (Diaspididae and Coccidae) and mealybugs (Pseudococcidae) to fumigation with a gas mixture of ethyl formate and carbon dioxide under normal atmospheric pressure or vacuum. Bull. Plant Prot. Japan..

[20] Griffin M.J., Jamieson L.E., Chhagan A., Page-Weir N.E.M., Poulton J., Davis V.A., Zulhendri F., Connolly P.G. (2013). The potential of ethyl formate + carbon dioxide to control a range of horticultural pests. N. Z. Plant Prot..

[21] Agarwal M., Ren Y., Newman J., Learmonth S. (2015). Ethyl formate: A potential disinfestation treatment of Eucalyptus weevil (*Gonipterus platensis*) (*Coleoptera: Curculionidae*) in apples. J. Econ. Entomol..

[22] Yang J.O., Park Y.R., Hyun I.H., Kim G.H., Kim B.S., Lee B.H., Ren Y.L. (2016). A combination treatment using ethyl formate and phosphine to control *Planococcus citri* (*Hemiptera: Pseudococcidae*) on pineapples. J. Econ. Entomol..

[23] Lee B.H., Kim H.M., Kim B.S., Yang J.O., Moon Y.M., Ren Y.L. (2016). Evaluation of the synergistic effect between ethyl formate and phosphine for control of *Aphis gossypii* (*Homoptera: Aphididae*). J. Econ. Entomol..

[24] Lee B.H., Park C.G., Yang J.O., Lee S.E. (2018). Concurrent application of ethyl formate and 1-methylcyclopropene to control *Tetranychus urticae* on exported sweet persimmons (*Diospyros Kaki* Thunb. ‘Fuyu’). Entomol. Res..

[25] Lee J.S., Kim H.K., Kyung Y.J., Park G.H., Lee B.H., Yang J.O., Koo H.N., Kim G.H. (2018). Fumigation activity of ethyl formate and phosphine against *Tetranychus urticae* (*Acari: Tetranychidae*) on imported sweet pumpkin. J. Econ. Entomol..

[26] Kyung Y.J., Kim H.K., Cho S.W., Kim B.S., Yang J.O., Koo H.N., Kim G.H. (2019). Comparison of the efficacy and phytotoxicity of phosphine and ethyl formate for controlling *Pseudococcus longispinus* (*Hemiptera: Pseudococcidae*) and *Pseudococcus orchidicola* in imported foliage nursery plants. J. Econ. Entomol..

[27] Choi J., Lim E., Park M.G., Cha W. (2020). Assessing the Retest Reliability of Prefrontal EEG Markers of Brain Rhythm Slowing in the Eyes-Closed Resting State. Clin. EEG Neurosci..

[28] Kwon T.H., Kim D.B., Kim K.Y., Park M.G., Roh G.H., Lee B.H. (2021). Scale-up ethyl formate fumigation to replace methyl bromide on traded mushroom to disinfest mushroom fly (*Lycoriella mali*). Appl. Biol. Chem..

[29] Kwon T.H., Park C.G., Lee B.H., Zarders D.R., Roh G.H., Kendra P.E., Cha D.H. (2021). Ethyl formate fumigation and ethyl formate plus cold treatment combination as potential phytosanitary quarantine treatments of *Drosophila suzukii* in blueberries. J. Asia-Pac. Entomol..

[30] Kim D.B., Kim K.W., Park M.G., Roh G.H., Cha D.H., Lee B.H. (2021). New feasible quarantine disinfestation using ethyl formte for termites and ants on imported lumber. J. Asia-Pac. Entomol..

[31] Kim D.B., Kwon T.H., Park M.G., Kim K.W., Cha D.H., Lee B.H. (2022). Ethyl formate-based quarantine treatment for exotic ants and termites in imported rubber plants and stone products. Appl. Sci..

[32] Kwon T.H., Park C.G., Lee B.H., Jeong I.H., Lee S.E. (2022). A New Approach: Ethyl Formate Fumigation to Control *Bemisia tabaci* (*Hemiptera: Aleyrodidae*) in a Yellow Melon Vinyl House. Appl. Sci..

[33] Song S.S., Oh H.K., Motoyama N. (1995). Insecticide resistance mechanism in the spiraea aphid, *Aphis citricola* (van der Goot). Korean J. Appl. Entomol..

[34] Ren Y.L., Lee B.H., Padovan B. (2011). Penetration of methyl bromide, sulfuryl fluoride, ethanedinitrile and phosphine into timber blocks and the sorption rate of the fumigants. J. Stored Prod. Res..

[35] Renkema J.M., Krey K., Devkota S., Liburd O.E., Funderburk J. (2020). Efficacy of insecticides for season-long control of thrips (*Thysanoptera: Thripidae*) in winter strawberries in Florida. Crop Prot..

[36] Wang Z., Gong Y., Jin G., Li B., Chen J., Kang Z., Zhu L., Gao Y., Reitz S., Wei S. (2016). Field-evolved resistance to insecticides in the invasive western flower thrips *Frankliniella occidentalis* (Pergande) (*Thysanoptera: Thripidae*) in China. Pest Manag. Sci..

[37] Kumar V., Kakkar G., McKenzie C.L., Seal D.R., Osborne L.S., Sonia Soloneski S., Marcelo Larramendy M. (2013). An Overview of Chilli Thrips, *Scirtothrips dorsalis* (*Thysanoptera: Thripidae*) Biology, Distribution and Management. Weed and Pest Control—Conventional and New Challenges.

[38] Bikoba V.N., Pupin F., Biasi W.V., Rutaganira F.U., Mitcham E.J. (2019). Use of Ethyl formate fumigation to control adult bean thrips in navel oranges. J. Econ. Entomol..

[39] Aharoni Y., Stewart J.K., Guadagni D.G., Mon T.R. (1980). Thrips mortality and strawberry quality after vacuum fumigation with acetaldehyde or ethyl formate. J. Am. Soc. Hortic. Sci..

[40] Pupin F., Bikoba V., Biasi W.B., Pedroso G.M., Ouyang Y., Grafton-Cardwell E.E., Mitcham E.J. (2013). Postharvest control of western flower thrips (*Thysanoptera: Thripidae*) and California red scale (*Hemiptera: Diaspididae*) with ethyl formate and its impact on citrus fruit quality. J. Econ. Entomol..

[41] van Epenhuijsen C.W., Somerfield K.G., Hedderley D.I., Brash D.W. (2007). Efficacy of ethyl formate and ethyl acetate for the control of onion thrips (*Thrips tabaci*). N. Z. J. Crop Hort. Sci..

[42] Slot M., Nardwattanawong T., Hernández G.G., Bueno A., Riederer M., Winter K. (2021). Large differences in leaf cuticle conductance and its temperature response among 24 tropical tree species from across a rainfall gradient. New Phytol..

[43] Berardini N., Fezer R., Conrad J., Beifuss U., Carle R., Schieber A. (2005). Screening of mango (*Mangifera indica* L.) cultivars for their contents of flavonol O- and xanthone C-glycosides, anthocyanins, and pectin. J. Agric. Food Chem..

[44] Lee M., Nam D.E., Kim O.K., Shim T.J., Kim J.H., Lee J. (2014). Anti-obesity effects of African mango (*Irvingia gabonesis*, IGOB 131TM) extract in leptin-deficient obese mice. J. Korean Soc. Food. Sci. Nutr..

